# Underexplored microbial metabolisms for enhanced nutrient recycling in agricultural soils

**DOI:** 10.3934/microbiol.2017.4.826

**Published:** 2017-10-13

**Authors:** Arnab Bhowmik, Mara Cloutier, Emily Ball, Mary Ann Bruns

**Affiliations:** 1 Department of Ecosystem Science and Management, The Pennsylvania State University, University Park, PA 16802, USA; 2 Dual-Title Graduate Program in Biogeochemistry, The Pennsylvania State University, University Park, PA 16802, USA; 3 Intercollege Graduate Degree Program in Ecology, The Pennsylvania State University, University Park, PA 16802, USA

**Keywords:** microbial metabolisms, redox potential, reduced tillage, cover cropping, organic amendments, efficient biological nutrient cycling, greenhouse gas, hydrogen consumption, atypical *nosZ*, *nrfA*, *euknr* gene

## Abstract

Worldwide, arable soils have been degraded through erosion and exhaustive cultivation, and substantial proportions of fertilizer nutrients are not taken up by crops. A central challenge in agriculture is to understand how soils and resident microbial communities can be managed to deliver nutrients to crops more efficiently with minimal losses to the environment. Throughout much of the twentieth century, intensive farming has caused substantial loss of organic matter and soil biological function. Today, more farmers recognize the importance of protecting soils and restoring organic matter through reduced tillage, diversified crop rotation, cover cropping, and increased organic amendments. Such management practices are expected to foster soil conditions more similar to those of undisturbed, native plant-soil systems by restoring soil biophysical integrity and re-establishing plant-microbe interactions that retain and recycle nutrients. Soil conditions which could contribute to desirable shifts in microbial metabolic processes include lower redox potentials, more diverse biogeochemical gradients, higher concentrations of labile carbon, and enrichment of carbon dioxide (CO_2_) and hydrogen gas (H_2_) in soil pores. This paper reviews recent literature on generalized and specific microbial processes that could become more operational once soils are no longer subjected to intensive tillage and organic matter depletion. These processes include heterotrophic assimilation of CO_2_; utilization of H_2_ as electron donor or reactant; and more diversified nitrogen uptake and dissimilation pathways. Despite knowledge of these processes occurring in laboratory studies, they have received little attention for their potential to affect nutrient and energy flows in soils. This paper explores how soil microbial processes could contribute to in situ nutrient retention, recycling, and crop uptake in agricultural soils managed for improved biological function.

## 1. Introduction

Native soil ecosystems have been converted for agricultural use since the dawn of human civilization. During the past century, global food demands have intensified land conversion, as well as use of fertilizers, irrigation, and mechanization [1]. Modern agriculture is dominated by large-scale, continuously mono-cropped fields that have incurred significant losses of soil and organic matter and require increasing amounts of fertilizers [2],[3]. Reliance on synthetic fertilizer has increased due to decoupling of crop and livestock production and less use of manures and legume rotations to restore soil fertility. On a worldwide basis, less than 50% of fertilizer nitrogen (N), regardless of source, is taken up by crops [4]. Nutrient imbalances are exacerbated as livestock production becomes more concentrated, resulting in further losses of unused reactive N to the environment [5].

While modern agriculture has helped address the daunting challenges of increased population and food demand, it has also led to deterioration of the soil's capacity to sustain plant and microbial biodiversity and perform ecosystem services [6],[7]. Native soils contain accumulated organic matter from decades to centuries of successional vegetation and decomposed litter, as well as intricate root-microbial networks belowground. Conversion of native soils to agriculture destroys the biological linkages between roots, mycelial networks, and interacting microorganisms, thus rendering soils more vulnerable to erosion [8]. Continuous agriculture precludes most plant residues from being returned to the soil, and repeated tillage further depletes soil organic matter through physical disruption and oxidation [9].

Awareness is growing, however, that a sustainable food supply calls for reversing decades of soil erosion and organic matter loss. More farmers are attempting to achieve this by reducing tillage, rotating crops, cover cropping, and returning more organic amendments to soils [3],[10],[11],[12]. Reduced- or no-tillage helps restore soil biophysical integrity and stabilizes microbial habitats to facilitate nutrient exchanges among microbes and between microbes and plants. Less disturbed soils may support development of lower soil oxidation-reduction potentials to enable microbial metabolic diversification. Crop rotations and cover cropping introduce a wider variety of organic compounds through greater root densities. Such management practices could foster adaptive microbial diversity in soils for better nutrient reutilization and fewer losses to the environment [13].

**Figure 1.**
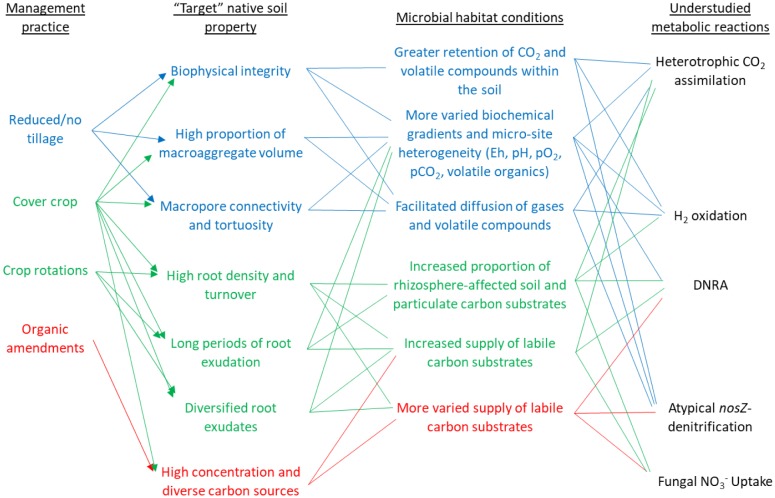
Schematic of agricultural management practices that aim to re-establish more native soil properties and create habitat conditions conducive to the microbial metabolisms highlighted in this review: heterotrophic CO_2_ assimilation, H_2_ oxidation, dissimilatory nitrate reduction to ammonium (DNRA), non-denitrifier N_2_O reduction, and fungal NO_3_^−^ uptake. Respective colors of text, connecting arrows, and lines are blue (for physical structure improvement); green (for increased plant inputs); and red (for more diverse organic inputs).

This paper highlights beneficial microbial metabolisms that could become more operational once soils are no longer subjected to intensive tillage and organic matter depletion. It describes how management-induced soil conditions (i.e., improved physical structure, higher organic matter content) could promote such microbial processes as heterotrophic CO_2_ consumption, H_2_ utilization, and diversified N respiratory pathways in soils (Figure 1). The rationale for this review is that biologically based agricultural management is expected to improve soil microbial habitat and increase microbial growth and diversity. When promoted in agricultural soils through management, these microbial processes could speed soil organic carbon (C) accretion, increase nutrient reuse, and reduce N losses to the environment.

## 2. Heterotrophic CO_2_ Consumption

Most soil microorganisms are aerobic heterotrophs and obtain their cell material and energy from reduced C atoms in organic matter. Soil microbes have estimated C use efficiencies (CUEs) ranging from 30 to 50%, with aerobic heterotrophic metabolism resulting in 50 to 70% of substrate-C being released as CO_2_ [14]. It has long been recognized that heterotrophic growth is enhanced in the presence of CO_2_ [15]. Growth enhancement by CO_2_ is attributable at least in part to the carboxylation of pyruvate and phosphoenolpyruvate (forming oxaloacetate) in anaplerotic reactions of the tricarboxylic acid cycle [16]. Anaplerotic uptake of CO_2_ is unlikely to be limited in undisturbed soils, where partial pressures of CO_2_ in soil pores can be up to 1000 times higher than they are in the atmosphere. This general metabolic process, however, could be affected when physical disruption of soils during plow tillage causes release of large amounts of soil CO_2_ to the atmosphere [17].

Microbial assimilation of CO_2_ can therefore result from heterotrophic as well as autotrophic metabolisms in soils. In mesocosm experiments using agricultural soils, CO_2_ assimilation accounted for 1–8.6% of total microbial biomass [18]. In pure culture studies with common heterotrophic soil bacteria (e.g., *Pseudomonas putida*), between 1.4–6.5% of cellular biomass can result from CO_2_ assimilation [19]. During studies of CO_2_ uptake by other heterotrophic bacteria, Roslev and coauthors showed strong correlations between growth and CO_2_ assimilation, with CO_2_-C mainly recovered in bacterial lipids [19]. In studies using stable isotope enrichments of soil, added ^13^C was recovered in amino acids, amino sugars and fatty acids of bacteria and actinomycetes, demonstrating the ability of microbes to incorporate CO_2_-C into multiple cellular compounds [20],[21].

In addition to generalized anaplerotic uptake, CO_2_ can also be consumed as a reactant during fermentative metabolisms. Several heterotrophic bacterial species in the phylum Firmicutes (e.g., *Clostridium spp._,_ Ruminococcus spp.* and *Butyribacterium* *rettgeri*) produce acetate from two CO_2_ molecules [22],[23]. Although acetogenic reactions in soils would be expected to occur only under suboxic or anoxic conditions, anaplerotic reactions by heterotrophs occur more widely. Moreover, CO_2_ assimilation can be stimulated by increased H_2_ availability. A recent study by Jones et al. [24] observed CUE close to 100% when *Clostridium spp.* were grown as mixotrophs using H_2_ as a reducing agent during fermentation. Likewise, CUE increased for other known acetogens like *Eubacterium limosum* and *Moorella thermoacetica* during fermentation with H_2_ additions [24].

Heterotrophic assimilation of extracellular CO_2_ could therefore increase CUE of soil microbial communities, speed accretion of microbial biomass and organic matter, and decrease greenhouse CO_2_ losses to the atmosphere as a greenhouse gas. This process may play an increasingly pivotal role in global C cycles as temperatures and respiration increase. Thus, understanding CO_2_ assimilation by heterotrophic microbes and the environmental cues that stimulate it may become more critical for maintaining biological functions in agricultural soils.

## 3. Utilization of H_2_

Dihydrogen gas, H_2_, is a widely available source of energy and reducing power for microorganisms that can utilize it under suitable conditions. Despite low-energy yields from coupling H_2_ oxidation with O_2_ reduction, H_2_ in soils may be important for maintaining viability of microbes with depleted C supplies [25],[26]. It has been estimated that for every gram of soil, oxidation of available H_2_ in soils could provide enough energy to maintain the viability of 10^7^ starved bacteria [27]. Analogous to substantial losses of soil CO_2_ observed after tillage, H_2_ losses also would be expected to occur as a result of physical disturbance. Although comparisons of H_2_ partial pressures in tilled and undisturbed soil have yet to be reported in the literature, it is reasonable to propose that H_2_ availability to resident microorganisms is higher and more consistent when soils remain undisturbed.

Many soils act as net sinks for H_2_, and it has been estimated that approximately 88 ± 11 Tg ha^−1^ of atmospheric H_2_ is taken up by soils each year [28]. Uptake of atmospheric H_2_ occurs at the soil-air interface, with H_2_ having a tropospheric half-life of ca. 1.4 yr [28] at an estimated concentration of 530 ppb [29]. Consumption of H_2_ is also a common metabolic reaction in rhizospheres of legumes, where symbiotic rhizobia produce H_2_ as a byproduct of N_2_ fixation [29],[30]. Concentrations of H_2_ at soil-nodule interfaces can be up to 20,000 times higher in the rhizosphere compared to the troposphere. Steep gradients in H_2_ concentrations occur with distance from nodules (decrease to sub-atmospheric levels within 4.5 cm from nodule), and measurable H_2_ consumption rates in soil have been observed [31],[32],[33].

Bacteria that use extracellular H_2_ as a reducing agent occur in diverse phyla [26],[34],[35],[36]. These bacteria can further be categorized into different groups based on their affinity for H_2_, which is dependent upon the specific [Ni-Fe] hydrogenase enzyme used by the bacteria. Group 1 [Ni-Fe] hydrogenases are membrane-bound enzymes that generally belong to species within the Proteobacteria phylum and are characterized as having a low-affinity for H_2_ [27]. In contrast, high-affinity hydrogenases can be either membrane bound or referred to as “abiontic hydrogenases” (exoenzymes), which can oxidize H_2_ at low concentrations [27]. Thus, it is hypothesized that H_2_ concentrations can influence the activity of different hydrogenase enzymes, specifically at the soil-atmosphere and soil-root nodule interfaces, which could favor and select for specific H_2_-oxidizing bacteria.

Oxygen serves as an important electron acceptor for H_2_ oxidation. In pure culture studies, *Actinobacteria* spp. were unable to oxidize H_2_ under anoxic conditions whereas H_2_ oxidation was stimulated as oxygen availability increased [27]. Other energy-yielding H_2_ oxidation reactions also could occur along gradients and interfaces, specifically the “Knallgas” reaction, where electrons are transferred between H_2_ and O_2_ to form H_2_O in oxic/suboxic zones [27]. Thus, H_2_ utilization expands energy supplies when reduced C sources become limiting. Other microorganisms under fermentative conditions can metabolize H_2_ and CO_2_ simultaneously [22], such as the acetogen *Acetobacterium* *woodii* [37]. *Clostridium* *themoaceticum* can also grow with either H_2_ or CO_2_, but the growth of *C*. *themoaceticum* was highest when both H_2_ and CO_2_ were supplied [38].

Mixotrophic and syntrophic growth strategies are also recognized as means by which some bacteria can enhance growth. *Mycobacterium smegmatis*, for example, can co-oxidize H_2_ and organic compounds simultaneously. If H_2_ is the sole electron donor, *M. smegmatis* growth is impeded [27]. Syntrophy can rely on transfers of H_2_ between different taxa. An example is the relationship between *Desulfovibrio alaskensis* and *Syntrophomonas wolfei.* Growth of *S. wolfei*, which was inhibited by the accumulation of H_2_, was increased in the presence of bacteria that oxidize H_2_, such as *D. alaskensis* [39]. In fact, expression of the [Ni-Fe] hydrogenase by *D. alaskensis* under syntrophic growth with *S. wolfei* was over 40 times higher than its expression when *D. alaskensis* was grown axenically [39], suggesting that the relationship stimulates H_2_ oxidation by *D. alaskensis*.

Expression of [Ni-Fe] hydrogenase enzymes by mixotrophs also may be increased during periods of energy limitation (i.e. oligotrophic environments). Under C limitations, *M. smegmatis* can upregulate the group 5 [Ni-Fe] hydrogenase enzyme and downregulate other hydrogenase enzymes that are not associated with H_2_ oxidation. Thus, it has been hypothesized that microbes will supplement their energy requirements by oxidizing H_2_ during periods of dormancy. Gene knockout studies have demonstrated the importance of H_2_ oxidation for viability of dormant cells of *M. smegmatis* and *Streptomyces avermitilis* [36]. It has been estimated that 95–99.9% of all microbial cells in soils are dormant [27], but it is not known what proportion of those cells might depend on the oxidation of H_2_ during those periods. Still, this information indicates that H_2_-oxidizing metabolic reactions are important in helping maintain microbial activity and diversity in soils [27]. In undisturbed soils, H_2_ can be recycled to keep soil microorganisms in more active states, thus serving to reduce lag times for mineralization of newly added organic substrates and facilitating release of inorganic nutrients to plants.

The study of H_2_ oxidation in soils has focused on the rhizosphere, where steep concentration gradients exist due to H_2_ release during N_2_ fixation by rhizobia [33]. It is likely that H_2_ oxidation occurs in other soil microhabitats, as evidenced by H_2_ uptake measurements [40],[41],[42], but much more needs to be learned about how soil management affects microbial use of H_2_ as an energy source for enhanced soil biological function. A potential concern in agroecosystems is the use of H_2_ by methanogens to reduce CO_2_ to methane (CH_4_). Supplementing agricultural soils with cow manure, for example, results in increased methanogen abundance and CH_4_ production [43]. In fact, manure addition can change the relationship between CH_4_ consumption and production in soils so that more CH_4_ is produced than consumed [43]. On the other hand, increased methanogenesis could be counterbalanced by more methanotrophic consumption of CH_4_ in rhizosphere soils. Therefore, development of soil microenvironments to foster bacterial consumption of CH_4_, CO_2_, and H_2_ could be one means to enhance nutrient use efficiency. It is reasonable to hypothesize that soils having varied microsites and redox potentials would possess greater gas uptake capacities than highly disturbed and degraded soils. Indeed, measurement of gas uptake capacity has the potential to be developed as an indicator of soil biological function.

Studying relationships between microbial metabolisms, gas production, and soil management remain problematic due to the spatial heterogeneity of microbial assemblages and microhabitats. Historically soil microbiological methods have involved removing samples from intact pedons, sieving/mixing, and co-mingling organisms that have had no spatial or physiological relationships whatsoever in the original soil. Therefore, a prerequisite for learning about relationships between microbial metabolisms and soil management will be to devise reliable methods for the study of gas exchange in intact soils. To test hypotheses regarding the relationships between gas metabolism and soil structural integrity, analysis methods enabling assessment of in situ metabolic processes will need to be developed and applied.

## 4. Diversified N Transformation Pathways

Fertilized soils are major sources of reactive N in the environment, and the many possible fates of nitrate (NO_3_-N) make it the most pivotal reactive N species in soil. Since NO_3_^−^ is a soluble anion that is repelled by negatively charged sites on soil particles, it is highly mobile and transported readily through soil by mass flow. If not assimilated into crops or soil microorganisms, NO_3_^−^ can be leached readily through the soil profile, lost in runoff, or denitrified (dissimilated) and lost to the atmosphere as different gaseous N species. While losses of inert N_2_ gas contribute to inefficient N use, they do not contribute directly to the greenhouse effect. Soil N losses as N_2_O, however, are environmentally more problematic, since N_2_O is 300 times more potent than CO_2_ as a greenhouse gas and speeds depletion of ozone [43]. In the United States, agriculture accounts for the majority (i.e., 75–80%) of anthropogenic N_2_O emissions, with fertilized soils and livestock wastes contributing about 60% and 30% of that total [4]. Lowering net N_2_O emissions is therefore crucial for mitigating agriculture's impact on global warming.

**Figure 2.**
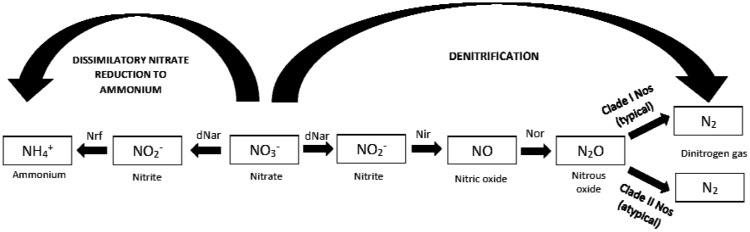
A depiction of respiratory nitrogen (N)-reducing enzymes involved in dissimilatory nitrate reduction to ammonium viz. dNar (dissimilatory nitrate reductase) and Nrf (cytochrome c nitrite reductase) and denitrification viz. dNar, Nir (nitrite reductase), Nor (nitric oxide reductase) and Nos (nitrous oxide reductase).

Heterotrophic (classic) denitrification is considered to be the main process responsible for N_2_O losses from most agricultural soils. Denitrification occurs when soils become wet, causing denitrifiers to switch from using O_2_ as an electron acceptor to NO_3_-N for anaerobic respiration. The classic denitrification sequence consists of stepwise N reductions from NO_3_^−^ to NO_2_^−^ to NO to N_2_O to N_2_ (Figure 2). Each step is carried out by a specific inducible enzyme, namely dissimilatory nitrate reductase (dNar), nitrite reductase (Nir), nitric oxide reductase (Nor) and nitrous oxide reductase (Nos) [44]. The entire sequence can take place within one organism possessing all requisite enzymes or by multiple organisms, necessitating the exchange of N intermediates. Denitrifiers are phylogenetically diverse, and many do not possess all enzymes needed to completely reduce NO_3_^−^ to N_2_ via N_2_O as an intermediate [45]. Thus, the main end products from denitrification (N_2_ or N_2_O) will depend not only on soil O_2_ content and electron donor availability, but also on microbial community structure and enzyme induction.

Although classic denitrification is the most well-studied N dissimilation process in soil, it is not the only means by which biological NO_3_^−^ reduction can occur. Some bacteria reduce NO_3_^−^ to NH_4_^+^ (Figure 2) in a process known as dissimilatory nitrate reduction to ammonium (DNRA). Soil conditions that favor DNRA over denitrification are poorly understood, but it is reasonable to expect that DNRA would extend N residence times in soils by preventing or delaying losses of N gases to the atmosphere. Reduction of N_2_O to N_2_ by non-denitrifiers is another process that could help lower net N_2_O emissions (Figure 2). Recent studies have described novel N_2_O-reducing enzymes present in a wide diversity of organisms which lack the enzymes to produce N_2_O [46]. Both DNRA and non-denitrifier N_2_O reduction represent N dissimilation pathways that could serve as N_2_O sinks in agricultural soils [47].

### 4.1. Dissimilatory nitrate reduction to ammonium

The DNRA process can be carried out by phylogenetically diverse bacteria and consists of two steps [44],[48]. Even though DNRA has been demonstrated in marine fungi and other marine eukaryotes [49], the discussion here focuses solely on DNRA by bacteria. The first of the two steps in DNRA is the initial reduction of NO_3_^−^-N to NO_2_^−^-N, which is similar to what occurs in classic denitrification. The first step can be catalyzed either by a periplasmic nitrate reductase complex (Nap) or a membrane-bound nitrate reductase (Nar) [50]. The second, more distinctive step is the reduction of NO_2_^−^-N to NH_4_^+^-N by cytochrome c nitrite reductase by formate (Nrf). From a thermodynamic standpoint, denitrification yields more energy than DNRA during respiration, but the inefficiencies of energy conservation during multiple denitrification steps makes the actual energy yield of DNRA higher than that of denitrification in pure cultures [51]. Most DNRA organisms use a non-membrane bound nitrite reductase for conversion of NO_2_^−^-N to NH_4_^+^-N and thus do not conserve energy but use the reaction as a sink for excess electrons. However, *Wolinella succinogenes* and other enteric species in the gammaproteobacteria use a membrane-bound nitrite reductase, which enables this reaction to be coupled to energy production [52]. In some cases, bacteria can perform DNRA for either electron disposal or to produce energy, as in *E. coli* [53]. An alternative benefit of DNRA is that the process is a means to detoxify NO_2_^−^ [54],[55],[56].

Recent advances in molecular detection of DNRA bacteria indicate surprisingly high genetic diversity and widespread distribution in the environment. Indeed, many enteric bacteria present in animal wastes (e.g., *E. coli*) are known to carry out this process using the DNRA, pentaheme cytochrome c nitrite reductase (Nrf), which requires Ca^2+^ for its activity [57],[58]. Enzymes other than Nrf may carry out dissimilative N reductions, such as multi-heme cytochrome proteins (e.g., octaheme tetrathionate reductase (Ota)) in *Shewanella oneidensis* [59]. However, validation of primers or probes for conserved sites in these genes has not yet been reported [60].

Genes encoding the Nrf enzymes provide genetic markers for DNRA populations and processes [61],[62],[63]. The study by Mohan et al. [61] was the first to describe primers for *nrfA* (a gene that encodes for the enzyme) based on extant DNA sequence accessions. These highly degenerate primers, which were designed by aligning amino acid sequences from *E. coli* K-12, *Sulfurospirillum deleyianum* and *Wolinella succinogenes*, amplified a 490-bp fragment of *nrfA*. Subsequently, it was found that these primers did not amplify *nrfA* from many DNRA organisms, which are now recognized to be quite phylogenetically diverse [48]. Thus, it was not surprising that the first published primer sets failed in other studies to yield amplicons from soil, despite the known occurrence of *nrfA* genes in common bacterial taxa found in soil.

With the objective of detecting other DNRA populations in a broader set of soils, Welsh et al. [65] designed a different forward primer for *nrfA*. The new forward primer was designed by aligning nucleotide regions that were conserved in at least 75% of the 474 newly reported NrfA amino acid sequences from the FUNGENE database (http://fungene.cme.msu.edu/ (accessed 10 July 2017)). When combined with one of the reverse primers of Mohan et al. [61], the new forward primer amplified a 269 bp fragment from soils collected from agricultural field sites in Illinois [64]. This new primer set was applied by Song et al. [63] to demonstrate the co-occurrence of higher DNRA rates and higher *nrfA* gene abundances in estuaries using stable isotope probing. That study was the first to show that *nrfA* gene abundance can be used as a potential genetic proxy for DNRA rates.

Another recent study using the primers of Welsh et al. [64] was conducted on DNA and cDNA in soils from tilled potato fields planted with different cover crops [65]. In that study, *nrfA* transcripts were detected in low-temperature soils during two successive winter seasons, but transcript abundances were not affected by cover crop type. In other studies, the amounts of available C in agricultural soils clearly altered the fate of NO_3_-N [10],[66]. Thus, C availability also is expected to influence the kinds of N dissimilation processes that occur in soils, and this has important practical implications for N conservation (Figure 1). Fazzolari et al. [55] reported that available C, rather than O_2_, was the main factor regulating DNRA in soils and that the effect of variable O_2_ concentrations depended on the ratios of available C to NO_3_-N.

Different C sources influence N dissimilation processes and their transient intermediates and end products. Although N_2_O is a known byproduct of DNRA activity [67], classic denitrification and nitrifier denitrification are still considered to be the major N_2_O-producing processes in oxic and suboxic soils, respectively [68],[69]. Thus, soil conditions favoring DNRA over denitrification are expected to have lower net N_2_O emissions [70]. Another factor that should favor DNRA over other N dissimilation processes is soil redox state [71]. Reduced or no-till management minimizes soil disturbance, improves soil water-holding capacity, and results in lower redox potentials than those observed in intensively tilled soils [72]. All these properties are expected to favor DNRA populations in soils [73]. Since other studies have shown that DNRA is less sensitive to fluctuating soil redox conditions [74],[75], DNRA activity may be easier to sustain in soils with appropriate management.

The fact that the DNRA process involves a transfer of three additional electrons (compared to denitrification during the reduction of NO_3_^−^-N) implies that DNRA will be favored when the supply of electron donors is high and when soil conditions are strongly reduced. While it is true that NH_4_^+^ products of DNRA are still subject to re-oxidation to NO_3_-N by nitrification, the specific N atoms involved will be held longer in soils due to their greater probability of being taken up by crops or assimilated by soil microorganisms before or after subsequent nitrification.

### 4.2. N_2_O reduction to N_2_ by non-denitrifiers

Another means by which net N_2_O emissions from soils could be lowered is by promoting activity of non-denitrifier populations that reduce N_2_O to N_2_ [76]. These populations employ N_2_O reductase [46] enzymes that differ from those used by complete denitrifiers in the final step of classical denitrification (Figure 2). Early molecular research assessing the potential of soil microbial communities to reduce N_2_O was based on the use of PCR primers for the *nosZ* gene [47]. However, substantial discrepancies have been observed in studies where shifts in *nosZ* communities were used to link N_2_O consumption rates during denitrification in an ecosystem, indicating the existence of one or more unaccounted sinks for N_2_O. More recently, two distinct groups of Nos proteins were identified based on expanded sequence databases for *nos*Z genes [46].

Most classic or complete denitrifiers belonging to alpha-, beta-, and gammaproteobacteria possess *nos*Z genes which group within Clade I, whereas other taxa possess atypical *nos*Z gene sequences grouping in Clade II [46]. Bioinformatics analyses have revealed that atypical *nosZ* sequences exhibit regulatory and functional components distinct from typical *nosZ* sequences [46]. So far, most environmental studies have used primers for typical *nosZ* genes to amplify environmental DNA to estimate abundance and activity of N_2_O-reducing populations [77],[78]. These primers, however, were found to be unsuitable for amplifying atypical *nosZ* genes found in other diverse taxa. Using a database of five *Anaeromyxobacter* genomes, Sanford et al. [46] developed primers for atypical *nos*Z to amplify 880-bp fragments from DNA of agricultural soils. They also demonstrated presence of atypical *nosZ* sequences in many additional taxa by screening 126 bacterial and 7 archaeal genomes. Jones et al. [76] subsequently developed primers for quantitative PCR assay for atypical *nosZ* gene abundances from environmental soil, freshwater sediments and activated sludge samples.

To date, half of the organisms screened for atypical *nosZ* genes are incomplete denitrifiers, including *Anaeromyxobacter* spp., which lack genes encoding nitrite reductase (Nir) enzymes [76]. Managing soils for lower N_2_O emissions would be aided by the activity of N_2_O-reducers like *Anaeromyxobacter* spp., which convert N_2_O to N_2_, thereby counteracting classic denitrification in the presence of high NO_3_^−^ concentrations [46]. Promoting populations of non-denitrifier N_2_O reducers could be an effective tool to address the potential for N_2_O production by fungi, which are more likely to flourish in no-till than in tilled soils. Although NO_3_-N reduction as an electron-accepting process is less common among fungi than it is among bacteria, the major fungal product is N_2_O rather than N_2_ [79]. Members of the Ascomycota, Basidiomycota and Mucoromycota are capable of using NO_3_^−^ as an electron acceptor, although this trait does not appear to be widely distributed within each of these phyla [80]. In a study by Maeda et al. [79], 70 of 207 fungal isolates tested were capable of producing N_2_O from NO_3_^−^, and this capability was most frequently observed in members of the order *Hypocreales*. Potential production of N_2_O by fungi in no-till soils thus could be offset by enhanced activity of non-denitrifier N_2_O reducers.

In an experiment conducted by Orellana et al. [81], atypical *nosZ* gene-carrying N_2_O reducers were dominated by *Anaeromyxobacter* spp. and outnumbered the typical *nosZ* carrying microorganisms in Illinois corn-belt soils. Atypical *nosZ*-containing bacteria appear to have diverse N metabolisms, with some, such as *Anaeromyxobacter* spp., possessing *nrfA* genes to potentially perform DNRA [82]. Other bacteria containing atypical *nosZ* genes, such as *Wolinella succinogens*, *Geobacillus thermodenitrificans*, and several *Bacillus* spp. from soils, have been shown to reduce N_2_O to N_2_. Different management practices could be used to shape the relative abundances and activities of populations with typical and atypical *nosZ* genes and affect the capacity of soil to act as a sink for N_2_O [83]. Atypical *nosZ* genes were found to be most prominent in soil samples taken directly from the field, whereas incubation experiments following nitrate and glucose addition resulted in a bottleneck effect and selected for typical *nosZ* carrying bacteria [84]. Estimation of the potential for N_2_O reduction by non-denitrifiers should aid in modeling efforts and better projections of N_2_O fluxes from agricultural management systems.

### 4.3. Fungal uptake of NO_3_-N

Molecular analyses of the soil N cycle have focused mostly on bacterial genes involved in dinitrogen fixation (*nif*), nitrification (*amo*) and the more downstream steps of nitrate respiration or dissimilation (*nir, nor, nos*) [85]. Since these genes are found in fewer, more specialized taxa, they have been considered more ecologically informative than the more widely distributed *nar* and *nap* genes for dissimilatory nitrate reduction [86]. Even more universal among bacteria are genes for assimilatory nitrate reduction (*nas*), which have been virtually ignored as a functional N cycle indicator. On the other hand, nitrate assimilation genes (*euknr*) in different fungal taxa might have potential as indicators of fungal N cycling activity in response to agricultural management.

The involvement of fungi in the N cycle of agricultural soils, although acknowledged, is not well-studied, and the distinctive functionalities of fungal saprotrophs and biotrophs add to the research challenge. Since fungal biomass is often (but not always) higher in no-till than in intensively tilled soils [87],[88], fungal contributions to N cycling are expected to become more important after soil disturbance ceases, due to less breakage of hyphal networks and less damage to mycorrhizal spores [89],[90]. Fungi would be expected to play greater roles in N cycling in surface residues and upper layers of no-till soils (0–5 cm), where larger increases in fungal biomass have been reported relative to deeper soils [90],[91]. Abundance and/or expression of fungal *euknr* could be an important indicator of fungal activity and nitrate uptake in less-disturbed soil strata.

Most fungi are capable of nitrate assimilation [92]. Fungi gained the ability to assimilate NO_3_^−^ following horizontal gene transfer of the *euknr* gene from oomycetes. Through the study of *Aspergillus nidulans* and *Neurospora crassa*, the enzymes and/or co-factors involved in nitrate assimilation and its regulation have been well characterized in ascomycetes [93]. Less is known about nitrate assimilation in basidiomycetes, although the enzyme for nitrate reductase, EukNR, is the same in each phylum [80].

Over the last 10 years several groups have created primer sets to specifically detect and amplify fungal nitrate reductases from soil communities. Nygren et al. [94] created primers to amplify nitrate reductases from Basidiomycetes, while Gorfer et al. [95] focused on nitrate reductase from ascomycetes, which are often the most abundant fungal taxa in agricultural soils. Neither of the primer sets were able to amplify nitrate reductases from all nitrate reductase-carrying ascomycetes or basidiomycetes. Primer sets designed by Gorfer et al. [95] showed a bias towards Pezizomycotina, while primers constructed by Nygren et al. [94] excluded species within the Russulacea family and *Amanita* genus. Still, these primer sets were able to amplify nitrate reductase sequences from either forest or agricultural soils. Despite the bias associated with fungal nitrate reductase primers, nitrate reductase sequence classifications and quantifications from agricultural soil were similar to fungal abundance and community structure based on ribosomal intergenic transcribed spacer sequencing [96].

Nitrate reductase fungal activity can be measured using several techniques; qPCR with appropriate primer sets or stable isotope probing and have been used to identify parameters that influence nitrate assimilation. Primers designed by Gorfer et al. [95] were used to quantify fungal expression of the nitrate reductase gene in microcosms using agricultural soil. Expression levels of *euknr* were only measurable after C addition, demonstrating the sensitivity of nitrate reductase activity in fungi towards C availability. Regulation of nitrate assimilation by C availability is most likely due to the energy intensive reduction processes required in NO_3_^−^ assimilation [97],[98],[99]. Identifying management practices that promote fungal growth and activity, therefore, could enhance NO_3_^−^ assimilation, with widespread implications for water and air quality, as less NO_3_^−^ would be lost through leaching or reduced to N_2_O by denitrifiers.

## 5. Facilitating Diverse Metabolic Processes Through Soil Management

**Figure 3.**
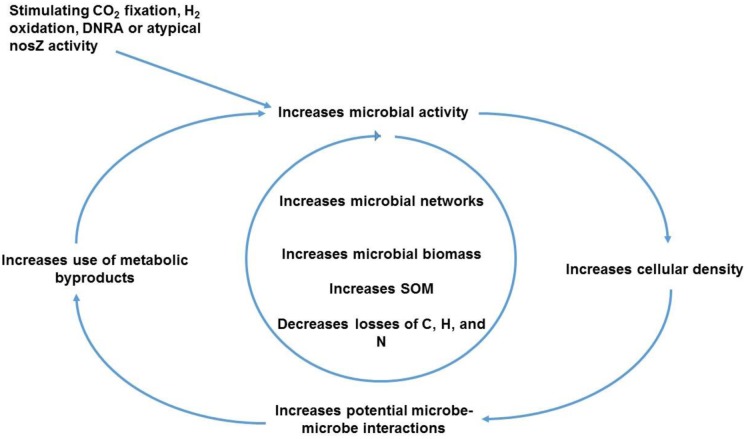
Microbial feedback responses resulting from conditions that stimulate underexplored diverse microbial metabolisms in agricultural soil.

Determining conditions that facilitate one or more of the aforementioned microbial processes in situ can be challenging due to the complexity and diversity of microbes, substrates, and soil properties. A recent study conducted by Raynaud and Nunan [100] in agricultural soils indicated that the potential of microbes to interact with each other was positively correlated with increased bacterial density. Agricultural management practices like minimizing tillage, integrating cover crops and diverse crop rotations along with animal amendments that help achieve “low-disturbance-higher-C” soils would promote higher bacterial density/activity, thereby stimulating metabolic reactions that could capitalize on wastes from other metabolic reactions (Figure 3).

At any given time, only 1–5% of bacteria in soil are thought to be actively metabolizing [25],[27]. Management strategies employing mixed cover species and crop rotations, which in turn increase microbial activity and metabolic diversity, will increase root densities and lengthen periods of live root activity. The C added to soil through various root exudates, rhizo-deposits, crop residues or animal amendments stimulates microbial activity, thereby increasing concentrations of reactants for CO_2_ assimilators, H_2_ oxidizers and populations that carry out DNRA. Higher DNRA rates, for example, were measured after addition of either rice straw or Chinese milk vetch residues to soils incubated under greenhouse conditions as compared to control soils without crop residues [101]. They also found DNRA rates to be positively correlated with the concentration of dissolved organic C during the incubation study. Addition of alfalfa residues yielded higher DNRA activity as compared to straw residues in another soil incubation experiment [102]. Labile C availability can also increase CO_2_ assimilation into organic matter by microbes [103]. It therefore follows that microbial growth and mineralization would be favored in the presence of higher CO_2_ concentrations in low-disturbance-higher C soil. Another example of a C amendment that can affect N transformations is biochar. In one study, adding biochar to soils resulted in changes in atypical *nosZ* transcripts and lower N_2_O emissions compared to unamended soils [77],[104].

In addition to C availability, NH_4_^+^ has been thought to regulate NO_3_^−^ assimilation by fungi. However, Inselsbacher et al. [105], reported that fungal NO_3_^−^ assimilation was unaffected by high concentrations of NH_4_^+^ in agricultural soil mesocosms assayed with stable isotopes. This led to their proposal that fungi would assimilate NO_3_^−^ faster than bacteria [105]. Increasing fungal growth and activity, especially glomalin production [106], also helps build soil structure, which in turn regulates gaseous exchange and oxidation-reduction potentials of microsites. Moreover, when soil disturbance is reduced, and fungi are allowed to establish viable networks, higher labile C supplies could stimulate fungal uptake of nitrate. Besides direct assimilation, another fate for NO_3_^−^ in fungal biomass is cytoplasmic translocation, since cytoplasm inside hyphal networks can move bidirectionally (i.e., to and from patches of high nutrient concentrations and to and from roots). Promoting viable fungi in soils therefore allows nitrates to be translocated in soil within “contained networks”, which would serve to enhance N retention and reduce leaching. Endophytic fungi could play an especially important role in improving N use efficiency by delivering NO_3_^−^ to plant roots.

The addition of animal wastes enriches soils with more anaerobic and microaerophilic organisms that are favored by lower redox conditions. Wastes from the gastrointestinal tracts of mammals contain enteric bacteria like *E. coli* that are capable of conducting DNRA [107]. It is therefore expected that soils receiving repeated manure additions would be enriched in DNRA bacteria. It is also known that at high soil pH, nitrite accumulation facilitates DNRA [108]. Agronomic practices which combine liming with manure additions can raise soil pH > 6.5 which could favor DNRA [109] and promote NO_2_^−^ accumulation [110]. However, more research needs to be conducted to assess the diversity and activity of DNRA organisms in manure amended soils.

Reduced-tillage soils managed with manures are expected to harbor more microaerophilic and anaerobic populations that could exploit syntrophic relationships facilitated by lower redox potentials. Restoring biophysical integrity and microsite heterogeneity through reduced soil disturbance should permit maintenance of higher CO_2_ and H_2_ concentrations over longer periods of time, thereby expanding the soil energy supply overall. Soil aggregation along with root architecture also controls soil bulk density as well as macropore connectivity and tortuosity. Identifying plant traits that provide favorable rhizosphere habitats could be important in designing crop management strategies to promote these microbial interactions. A study by Hansel et al. [111] demonstrated how anaerobic microenvironments within soil aggregates provide conditions that are conducive to both anaerobic- and aerobic-based metabolisms. Co-occurrence of aerobic and anaerobic microbial habitats due to microscale heterogeneity [111] could therefore promote the understudied microbial metabolisms highlighted in this paper.

## 6. Conclusions

In this review we have attempted to relate management practices intended to mimic native soil conditions with insights from the literature on CO_2_ assimilation, H_2_ oxidation, alternative N transformation pathways, and enhanced fungal involvement in N cycling. We have the properties of undisturbed, native soils as management targets for plant-soil systems that could result in less nutrient loss. At the same time, we acknowledge that native soils are not capable of delivering needed amounts of nutrients on a sustained basis for agricultural production as we know it. In order for agricultural systems to be assisted by soil microbial processes, appropriate plant choices, organic amendments, and soil management practices will be needed to establish soil conditions that permit sustained activity of diverse microbial metabolisms. We also acknowledge that soils which have undergone organic matter depletion for long periods will not return quickly to the conditions once extant in native soils. Nevertheless, less disturbed soils are expected to become more spatially and temporally heterogeneous eventually over time.

One of the approaches to assess the importance of specific microbial metabolisms in increasing nutrient cycling efficiency will be to couple next generation sequencing and metabolomics with biogeochemical process measurements applied to soils with well-characterized management histories and field records. Such studies will help to identify taxa that play critical roles in improving nutrient cycling function and relate these agricultural management practices. Deeper understanding of potential soil microbial metabolisms is needed so that agricultural soils can be managed in a more ecologically and environmentally sustainable manner. Gaining insights into conditions that lead to ecologically beneficial microbial metabolisms will help to align agricultural management practices with efficient nutrient cycling and lower environmental impact.

## References

[1] Phelps J, Carrasco LR, Webb EL (2013). Agricultural intensification escalates future conservation costs. P Natl Acad Sci USA.

[2] Lal R (2004). Soil carbon sequestration impacts on global climate change and food security. Science.

[3] West TO, Post WM (2002). Soil organic carbon sequestration rates by tillage and crop rotation. Soil Sci Soc Am J.

[4] Galloway JN, Dentener FJ, Capone DG (2004). Nitrogen cycles: past, present, and future. Biogeochemistry.

[5] Inselsbacher E (2009). The fate of inorganic nitrogen fertilizers in agricultural soils.

[6] Tilman DG, Cassman KG, Matson PA (2002). Agricultural sustainability and intensive production practices. Nature.

[7] Gomiero T (2016). Soil degradation, land scarcity and food security: Reviewing a complex challenge. Sustainability.

[8] Habig J, Swanepoel C (2015). Effects of conservation agriculture and fertilization on soil microbial diversity and activity. Environments.

[9] Conant RT, Easter M, Paustian K (2007). Impacts of periodic tillage on soil C stocks: A synthesis. Soil Till Res.

[10] Drinkwater LE, Wagoner P, Sarrantonio M (1998). Legume-based cropping systems have reduced carbon and nitrogen losses. Nature.

[11] McDaniel MD, Tiemann LK, Grandy AS (2014). Does agricultural crop diversity enhance soil microbial biomass and organic matter dynamics? A meta-analysis. Ecol Appl.

[12] Bhowmik A, Fortuna A, Cihacek LJ (2017). Potential carbon sequestration and nitrogen cycling in long-term organic management systems. Renew Agr Food Syst.

[13] Morriën E, Hannula SE, Snoek LB (2017). Soil networks become more connected and take up more carbon as nature restoration progresses. Nat Commun.

[14] Paul EA, Morris SJ, Bohm S, Lal R, Kimble JM, Follett RF (2001). The determination of soil C pool sizes and turnover rates: biophysical fractionation and tracers. Assessment Methods for Soil Carbon.

[15] Alexander M (1977). Introduction to soil microbiology.

[16] Krebs HA (1941). Carbon dioxide assimilation in heterotrophic organisms. Nature.

[17] Reicosky DC, Archer DW (2007). Moldboard plow tillage depth and short-term carbon dioxide release. Soil Till Res.

[18] Wu X, Ge T, Yuan H (2014). Changes in bacterial CO_2_ fixation with depth in agricultural soils. Appl Micobiol Biot.

[19] Roslev P, Larsen MB, Jørgensen D (2004). Use of heterotrophic CO_2_ assimilation as a measure of metabolic activity in planktonic and sessile bacteria. J Microbiol Meth.

[20] Miltner A, Richnow H, Kopinke F (2004). Assimilation of CO_2_ by soil microorganisms and transformation into soil organic matter. Org Geochem.

[21] Miltner A, Richnow H, Kopinke F (2005). Incorporation of carbon originating from CO_2_ into different compounds of soil microbial biomass and soil organic matter. Isot Environ Healt S.

[22] Bernalier A, Willems A, Leclerc M (1996). *Ruminococcus hydrogenotrophicus* sp. nov., a new H_2_/CO_2_-utilizing acetogenic bacterium isolated from human feces. Arch Microbiol.

[23] Barker HA, Kamen MD, Haas V (1945). Carbon dioxide utilization in the synthesis of acetic and butyric acids by *Butyribacterium rettgeri*. P Natl Acad Sci USA.

[24] Jones SW, Fast AG, Carlson ED (2016). CO_2_ fixation by anaerobic non-photosynthetic mixotrophy for improved carbon conversion. Nat Commun.

[25] Hobbie JE, Hobbie EA (2013). Microbes in nature are limited by carbon and energy: the starving-survival lifestyle in soil and consequences for estimating microbial rates. Front Microbiol.

[26] Meredith LK, Rao D, Bosak T (2014). Consumption of atmospheric hydrogen during the life cycle of soil-dwelling Actinobacteria. Env Microbiol Rep.

[27] Greening C, Constant P, Hards K (2015). Atmospheric hydrogen scavenging: from enzymes to ecosystems. Appl Environ Microb.

[28] Rhee TS, Brenninkmeijer CAM, Röckmann T (2006). The overwhelming role of soils in the global atmospheric hydrogen cycle. Atmos Chem Phys.

[29] Maimaiti J, Zhang Y, Yang J (2007). Isolation and characterization of hydrogen-oxidizing bacteria induced following exposure of soil to hydrogen gas and their impact on plant growth. Environ Microbiol.

[30] Piché-Choquette S, Tremblay J, Tringe SG (2016). H_2_-saturation of high affinity H_2_-oxidizing bacteria alters the ecological niche of soil microorganisms unevenly among taxonomic groups. PeerJ.

[31] La FJS, Focht DD (1983). Conservation in soil of H_2_ liberated from N_2_ fixation by Hup-nodules. Appl Environ Microb.

[32] Witty JF (1991). Microelectrode measurements of hydrogen concentrations and gradients in legume nodules. J Exp Bot.

[33] Dong Z, Layzell DB (2001). H_2_ oxidation, O_2_ uptake and CO_2_ fixation in hydrogen treated soils. Plant Soil.

[34] Tiso M, Schechter AN (2015). Nitrate reduction to nitrite, nitric oxide and ammonia by gut bacteria under physiological conditions. PloS One.

[35] Greening C, Berney M, Hards K (2014). A soil actinobacterium scavenges atmospheric H_2_ using two membrane-associated, oxygen-dependent [NiFe] hydrogenases. P Natl Acad Sci USA.

[36] Liot Q, Constant P (2016). Breathing air to save energy–new insights into the ecophysiological role of high-affinity [NiFe]-hydrogenase in *Streptomyces avermitilis*. MicrobiologyOpen.

[37] Braun K, Gottschalk G (1981). Effect of molecular hydrogen and carbon dioxide on chemo-organotrophic growth of *Acetobacterium woodii* and *Clostridium aceticum*. Arch Microbiol.

[38] Kellum R, Drake HL (1984). Effects of cultivation gas phase on hydrogenase of the acetogen *Clostridium thermoaceticum*. J Bacteriol.

[39] Krumholz LR, Bradstock P, Sheik CS (2015). Syntrophic growth of *Desulfovibrio alaskensis* requires genes for H_2_ and formate metabolism as well as those for flagellum and biofilm formation. Appl Environ Microb.

[40] Schmitt S, Hanselmann A, Wollschläger U (2009). Investigation of parameters controlling the soil sink of atmospheric molecular hydrogen. Tellus B.

[41] Smith-Downey NV, Randerson JT, Eiler JM (2006). Temperature and moisture dependence of soil H_2_ uptake measured in the laboratory. Geophys Res Lett.

[42] Constant P, Poissant L, Villemur R (2009). Tropospheric H_2_ budget and the response of its soil uptake under the changing environment. Sci Total Environ.

[43] Team CW (2014). Climate change 2014: Synthesis report. Contribution of working groups I, II and III to the fifth assessment of the Intergovernmental Panel on Climate Change.

[44] Zumft WG (1997). Cell biology and molecular basis of denitrification. Microbiol Mol Biol Rev.

[45] Dandie CE, Burton DL, Zebarth BJ (2007). Analysis of denitrification genes and comparison of *nosZ*, *cnorB* and 16S rDNA from culturable denitrifying bacteria in potato cropping systems. Syst Appl Microbiol.

[46] Sanford RA, Wagner DD, Wu Q (2012). Unexpected nondenitrifier nitrous oxide reductase gene diversity and abundance in soils. P Natl Acad Sci USA.

[47] Henry S, Bru D, Stres B (2006). Quantitative detection of the *nosZ* gene, encoding nitrous oxide reductase, and comparison of the abundances of 16S rRNA, *narG*, *nirK*, and *nosZ* genes in soils. Appl Environ Microb.

[48] Smith CJ, Nedwell DB, Dong LF (2007). Diversity and abundance of nitrate reductase genes (*narG* and *napA*), nitrite reductase genes (*nirS* and *nrfA*), and their transcripts in estuarine sediments. Appl Environ Microb.

[49] Kamp A, Høgslund S, Risgaard-Petersen N (2015). Nitrate storage and dissimilatory nitrate reduction by eukaryotic microbes. Front Microbiol.

[50] Simon J (2002). Enzymology and bioenergetics of respiratory nitrite ammonification. FEMS Microbiol Rev.

[51] Strohm TO, Griffin B, Zumft WG (2007). Growth yields in bacterial denitrification and nitrate ammonification. Appl Environ Microb.

[52] Kern M, Simon J (2008). Characterization of the NapGH quinol dehydrogenase complex involved in *Wolinella succinogenes* nitrate respiration. Mol Microbiol.

[53] Cole J (1996). Nitrate reduction to ammonia by enteric bacteria: redundancy, or a strategy for survival during oxygen starvation?. FEMS Microbiol Lett.

[54] Tiedje JM (1988). Ecology of denitrification and dissimilatory nitrate reduction to ammonium. Biol Anaerob Microorgan.

[55] Fazzolari É, Nicolardot B, Germon JC (1998). Simultaneous effects of increasing levels of glucose and oxygen partial pressures on denitrification and dissimilatory nitrate reduction to ammonium in repacked soil cores. Eur J Soil Biol.

[56] Tiedje JM, Sexstone AJ, Myrold DD (1982). Denitrification: ecological niches, competition and survival. Anton Leeuw.

[57] Simon J, Klotz MG (2013). Diversity and evolution of bioenergetic systems involved in microbial nitrogen compound transformations. BBA-Bioenergetics.

[58] Cunha CA, Macieira S, Dias JM (2003). Cytochrome c nitrite reductase from *Desulfovibrio desulfuricans* ATCC 27774 the relevance of the two calcium sites in the structure of the catalytic subunit (*nrfA*). J Biol Chem.

[59] Atkinson SJ, Mowat CG, Reid GA (2007). An octaheme c-type cytochrome from *Shewanella oneidensis* can reduce nitrite and hydroxylamine. FEBS Lett.

[60] Doyle RAS, Marritt SJ, Gwyer JD (2013). Contrasting catalytic profiles of multiheme nitrite reductases containing CxxCK heme-binding motifs. J Biol Inorg Chem.

[61] Mohan SB, Schmid M, Jetten M (2004). Detection and widespread distribution of the *nrfA* gene encoding nitrite reduction to ammonia, a short circuit in the biological nitrogen cycle that competes with denitrification. FEMS Microbiol Ecol.

[62] Kraft B, Strous M, Tegetmeyer HE (2011). Microbial nitrate respiration—genes, enzymes and environmental distribution. J Biotechnol.

[63] Song B, Lisa JA, Tobias CR (2014). Linking DNRA community structure and activity in a shallow lagoonal estuarine system. Front Microbiol.

[64] Welsh A, Chee-Sanford JC, Connor LM (2014). Refined NrfA phylogeny improves PCR-based nrfA gene detection. Appl Environ Microb.

[65] Tatti E, Goyer C, Zebarth BJ (2017). Over-winter dynamics of soil bacterial denitrifiers and nitrite ammonifiers influenced by crop residues with different carbon to nitrogen ratios. Appl Soil Ecol.

[66] Bhowmik A, Fortuna A, Cihacek LJ (2016). Use of biological indicators of soil health to estimate reactive nitrogen dynamics in long-term organic vegetable and pasture systems. Soil Biol Biochem.

[67] Stremińska MA, Felgate H, Rowley G (2012). Nitrous oxide production in soil isolates of nitrate-ammonifying bacteria. Env Microbiol Rep.

[68] Firestone MK, Davidson EA (1989). Microbiological basis of NO and N_2_O production and consumption in soil. Exchange of trace gases between terrestrial ecosystems and the atmosphere.

[69] Zhu X, Burger M, Doane TA (2013). Ammonia oxidation pathways and nitrifier denitrification are significant sources of N_2_O and NO under low oxygen availability. P Natl Acad Sci USA.

[70] Rütting T, Boeckx P, Müller C (2011). Assessment of the importance of dissimilatory nitrate reduction to ammonium for the terrestrial nitrogen cycle. Biogeosciences.

[71] Matheson FE, Nguyen ML, Cooper AB (2002). Fate of ^15^N-nitrate in unplanted, planted and harvested riparian wetland soil microcosms. Ecol Eng.

[72] Clay DE, Molina JAE, Clapp CE (1990). Soil tillage impact on the diurnal redox-potential cycle. Soil Sci Soc Am J.

[73] Yin SX, Chen D, Chen LM (2002). Dissimilatory nitrate reduction to ammonium and responsible microorganisms in two Chinese and Australian paddy soils. Soil Biol Biochem.

[74] Pett-Ridge J, Silver WL, Firestone MK (2006). Redox fluctuations frame microbial community impacts on N-cycling rates in a humid tropical forest soil. Biogeochemistry.

[75] Em VDB, Van DU, Abbas B (2015). Enrichment of DNRA bacteria in a continuous culture. Int Soc Micobiol Ecol.

[76] Jones CM, Graf DRH, Bru D (2013). The unaccounted yet abundant nitrous oxide-reducing microbial community: a potential nitrous oxide sink. Int Soc Micobiol Ecol.

[77] Harter J, Krause H, Schuettler S (2014). Linking N_2_O emissions from biochar-amended soil to the structure and function of the N-cycling microbial community. Int Soc Micobiol Ecol.

[78] Gao J, Xie Y, Jin H (2016). Nitrous oxide emission and denitrifier abundance in two agricultural soils amended with crop residues and urea in the north China plain. PloS One.

[79] Maeda K, Spor A, Edel-Hermann V (2015). N_2_O production, a widespread trait in fungi. Sci Rep.

[80] Gorfer M, Klaubauf S, Berger H, Marco D (2014). The fungal contribution to the nitrogen cycle in agricultural soils. Metagenomics of the Microbial Nitrogen Cycle: Theory, Methods and Applications.

[81] Orellana LH, Rodriguez-R LM, Higgins S (2014). Detecting nitrous oxide reductase (nosZ) genes in soil metagenomes: method development and implications for the nitrogen cycle. Mbio.

[82] Sanford RA, Cole JR, Tiedje JM (2002). Characterization and description of *Anaeromyxobacter dehalogenans* gen. nov., sp. nov., an aryl-halorespiring facultative anaerobic myxobacterium. Appl Environ Microb.

[83] Yoon S, Nissen S, Park D (2016). Nitrous oxide reduction kinetics distinguish bacteria harboring clade I NosZ from those harboring clade II NosZ. Appl Environ Microb.

[84] Coyotzi S, Doxey AC, Clark ID (2017). Agricultural soil denitrifiers possess extensive nitrite reductase gene diversity. Environ Microbiol.

[85] Thompson KA, Bent E, Abalos D (2016). Soil microbial communities as potential regulators of in situ N_2_O fluxes in annual and perennial cropping systems. Soil Biol Biochem.

[86] Bru D, Sarr A, Philippot L (2007). Relative abundances of proteobacterial membrane-bound and periplasmic nitrate reductases in selected environments. Appl Environ Microb.

[87] Frey SD, Elliott ET, Paustian K (1999). Bacterial and fungal abundance and biomass in conventional and no-tillage agroecosystems along two climatic gradients. Soil Biol Biochem.

[88] Helgason BL, Walley FL, Germida JJ (2009). Fungal and bacterial abundance in long-term no-till and intensive-till soils of the Northern Great Plains. Soil Sci Soc Am J.

[89] McGonigle TP, Miller MH (1996). Mycorrhizae, phosphorus absorption, and yield of maize in response to tillage. Soil Sci Soc Am J.

[90] Kabir Z (2005). Tillage or no-tillage: impact on mycorrhizae. Can J Plant Sci.

[91] Sipilä TP, Yrjälä K, Alakukku L (2012). Cross-site soil microbial communities under tillage regimes: fungistasis and microbial biomarkers. Appl Environ Microb.

[92] Slot JC, Hibbett DS (2007). Horizontal transfer of a nitrate assimilation gene cluster and ecological transitions in fungi: a phylogenetic study. PloS One.

[93] Marzluf GA (1997). Genetic regulation of nitrogen metabolism in the fungi. Microbiol Mol Biol Rev.

[94] Nygren CMR, Eberhardt U, Karlsson M (2008). Growth on nitrate and occurrence of nitrate reductase-encoding genes in a phylogenetically diverse range of ectomycorrhizal fungi. New Phytol.

[95] Gorfer M, Blumhoff M, Klaubauf S (2011). Community profiling and gene expression of fungal assimilatory nitrate reductases in agricultural soil. Int Soc Micobiol Ecol.

[96] Klaubauf S, Inselsbacher E, Zechmeister-Boltenstern S (2010). Molecular diversity of fungal communities in agricultural soils from Lower Austria. Fungal Divers.

[97] Hankinson O, Cove DJ (1974). Regulation of the pentose phosphate pathway in the fungus *Aspergillus nidulans.* The effect of growth with nitrate. J Biol Chem.

[98] Hankinson O (1974). Mutants of the pentose phosphate pathway in *Aspergillus nidulans*. J Bacteriol.

[99] Schinko T, Berger H, Lee W (2010). Transcriptome analysis of nitrate assimilation in *Aspergillus nidulans* reveals connections to nitric oxide metabolism. Mol Microbiol.

[100] Raynaud X, Nunan N (2014). Spatial ecology of bacteria at the microscale in soil. PloS One.

[101] Lu W, Zhang H, Min J (2015). Dissimilatory nitrate reduction to ammonium in a soil under greenhouse vegetable cultivation as affected by organic amendments. J Soils Sed.

[102] DeCatanzaro JB, Beauchamp EG, Drury CF (1987). Denitrification vs dissimilatory nitrate reduction in soil with alfalfa, straw, glucose and sulfide treatments. Soil Biol Biochem.

[103] Miltner A, Kopinke F, Kindler R (2005). Non-phototrophic CO_2_ fixation by soil microorganisms. Plant Soil.

[104] Harter J, Weigold P, El-Hadidi M (2016). Soil biochar amendment shapes the composition of N_2_O-reducing microbial communities. Sci Total Environ.

[105] Inselsbacher E, Hinko-Najera UN, Stange FC (2010). Short-term competition between crop plants and soil microbes for inorganic N fertilizer. Soil Biol Biochem.

[106] Bronick CJ, Lal R (2005). Soil structure and management: a review. Geoderma.

[107] Cole JA (1978). The rapid accumulation of large quantities of ammonia during nitrite reduction by *Escherichia coli*. FEMS Microbiol Lett.

[108] Burns LC, Stevens RJ, Laughlin RJ (1995). Determination of the simultaneous production and consumption of soil nitrite using 15N. Soil Biol Biochem.

[109] Stevens RJ, Laughlin RJ (1998). Measurement of nitrous oxide and di-nitrogen emissions from agricultural soils. Nutr Cycl Agroecosys.

[110] Venterea RT (2007). Nitrite-driven nitrous oxide production under aerobic soil conditions: kinetics and biochemical controls. Global Change Biol.

[111] Hansel CM, Fendorf S, Jardine PM (2008). Changes in bacterial and archaeal community structure and functional diversity along a geochemically variable soil profile. Appl Environ Microb.

